# Congenital Portal Vein Aneurysm Associated with Peliosis Hepatis and Intestinal Lymphangiectasia

**DOI:** 10.1155/2009/479264

**Published:** 2010-03-30

**Authors:** Zeynel Mungan, Binnur Pinarbasi, Baris Bakir, Mine Gulluoglu, Bulent Baran, Filiz Akyuz, Kadir Demir, Sabahattin Kaymakoglu

**Affiliations:** ^1^Department of Gastroenterohepatology, Istanbul Medical Faculty, Istanbul University, Millet Cad. 34390 Capa, Istanbul, Turkey; ^2^Department of Radiology, Istanbul Medical Faculty, Istanbul University, Istanbul, Turkey; ^3^Department of Pathology, Istanbul Medical Faculty, Istanbul University, Istanbul, Turkey

## Abstract

Portal vein aneurisym (PVA), peliosis hepatis (PH) and intestinal lymphangiectasia (IL) all are very uncommon entities. Herein, we presented a unique patient with these three rare entities who was admitted to our hospital because of portal hypertensive ascites rich in protein and lymphocyte. PVA was extrahepatic and associated with coronary vein aneurysm. Peliosis hepatis was of microscopic form. Lymphangiectasia was present in peritoneum and small intestine. Diagnoses of these rare entities were made by imaging techniques and histopathological findings. Patient also had hydronephrosis caused by ureteropelvic junction narrowing. Best of our knowledge, there is no such a case reported previously with the association of PVA, PH and IL. Therefore, we propose PVAPHIL syndrome to define this novel association.

## 1. Introduction

Portal vein aneurysm (PVA) is a very infrequent vascular abnormality and has been rarely reported. Less than 25 extrahepatic PVAs have thus far been reported. Of these 25 cases, only 5 have aneurysm diameter over 6 cm [1]. Herein, we present a case with giant congenital PVA with concomitant peliosis hepatis (PH) and intestinal lymphangiectasia (IL), which are also very rare entities. Moreover, our subject had hydronephrosis due to ureteropelvic junction narrowing. Association of all these entities has not been reported previously.

## 2. Case Report

A 36-year-old female with a two-month history of progressive abdominal swelling due to ascites and hepatosplenomegaly was admitted to our hospital. Her medical history was unremarkable except for an inguinal hernia operation. Physical examination showed a thin and slightly pale patient with ascites 3 cm above the umbilicus; the liver and spleen were palpable at 2 cm and 4 cm below the costal margin, respectively. Ascites fluid analysis was as follows: leukocyte count: 600/mm^3^ (neutrophile, 100/mm^3^; lymphocyte, 400/mm^3^), serum-to-ascites albumin gradient (SAAG): 2.5 g/dL, total protein: 2.4 g/dL, trygliceride: 48 mg/dl, lactate dehydrogenase (LDH): 147 IU/L, with concurrent serum total protein: 6.9 g/dL and LDH: 346 IU/L. Ascites fluid was not chylous. Acid fast smear, cytological examination, and bacterial culture of the ascitic fluid were negative. Complete blood count (CBC) revealed hypochromic microcytic anemia (hemoglobin concentration, 10.4 g/dL) and lymphocytopenia (900/mm^3^) and platelet and leukocyte counts were normal. Iron deficiency was present with ferritin 23 ng/mL and transferrin saturation less than 10%. Erythrocyte sedimentation rate (ESR) and C-reactive protein (CRP) concentration were elevated at 49 mm/h and 23 mg/L, respectively. Liver function tests and coagulation parameters were normal. Other biochemical investigations were unremarkable except for hypogammaglobulinemia (0.68 g/dL). The patient was consulted with hematology for elevated ESR, hypogammaglobulinemia, and lymphocytopenia. Bone marrow aspiration cytology and biopsy revealed no significant pathology. The patient did not carry the JAK2 mutation. Serologic tests for HIV, hepatitis B and C were negative. Tumor markers including alpha-fetoprotein, CEA, CA 19-9, CA 15-3, and beta-HCG were also normal. She did not have any renal problem (malignancy, chronic or acute renal failure) which may cause ascites and glomerular filtration rate was 110 mL/minute. Urine sediment was normal. Gynecologic examination was normal. We did not find any cardiac pathology which can cause ascites and there were no findings about pericarditis in echocardiographic examination and thorax computerized tomography. The portal and splenic blood flow volumes were 5703 mL/min and 147.5 mL/min, respectively, in Doppler ultrasonography. Thus, the patient was diagnosed to have portal hypertension and further investigations were planned for the underlying etiology. 

**Figure 1:**
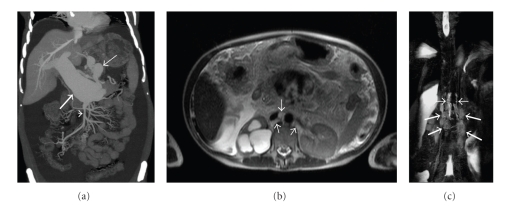
(a) Coronal plane maximum-intensity-projection reformatted CT image shows the fusiform dilatation of the main portal vein (thick arrow). There is also marked dilatation and tortuosity at coronary vein (thin arrow). Note that the caliber of superior mesenteric vein is normal (arrowhead). (b) Axial half-Fourier acquisition single-shot turbo spin-echo (HASTE) MR image demonstrates thick lymphatic channels in the retrocrural space (arrows). Also, there is hydnonephrosis at the right kidney. (c) MR-Lymphangiography image (a half-Fourier single-shot turbo spin-echo 2D sequence with breath-hold technique with maximum intensity projection (MIP)) demonstrates two tortuous tubular structures on each side of the aorta representing dilated cisterna chyli (thin arrows). The meshwork of saccular lymphatic channels in the lumbar region inferior to the cisterna represents the dilated lumbar lymphatics (thick arrows).

**Figure 2:**
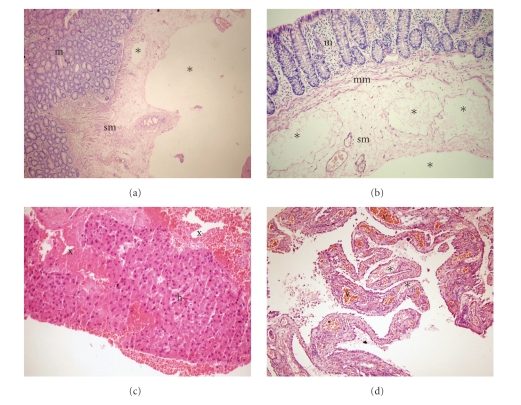
(a), (b) Edema of the submucosa and muscularis mucosa, and dilatation of the submucosal lymphatic vessels (asterixes). (c) Blood filled cavities (x) in the liver biopsy, (d) peritoneal biopsy displaying dilated lymphatic (asterixes) and capillary (v) vessels. m: mucosa, sm: submucosa, mm: muscularis mucosa, h: hepatocytes.

**Figure 3:**
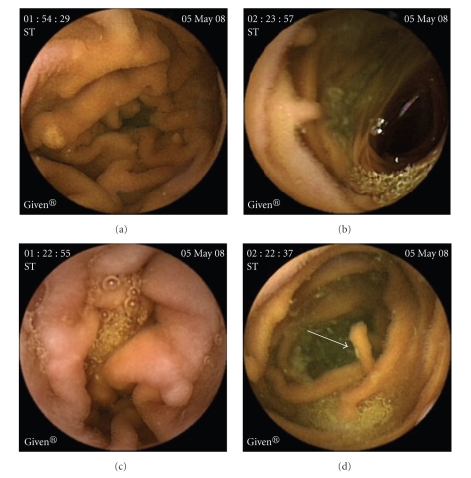
Diffuse polypoid lesions sized 2–5 mm throughout the small intestine in capsule endoscopy.

On abdominal computerized tomography (CT) a giant portal vein aneurysm and dilated coronary vein (Figure 1(a)) was present. Also, grade IV right hydronephrosis due to stricture at the ureteropelvic junction (Figure 1(b)) was detected. On CT angiography, there was no thrombus in the portal vein; superior mesenteric and splenic veins were at normal caliber. Gastroduodenoscopy and colonoscopy revealed minimally dilated distal esophageal veins, widespread gastroduodenal, and colonic polypoid lesions. In the histopathological examination of the endoscopic biopsy samples retrieved from the large bowel mucosa, dilated submucosal lymphatic vessels as well as mucosal and submucosal edema (Figures 2(a), 2(b)) were evident. Mucosal biopsies of gastrointestinal tract demonstrated mild reflux esophagitis, severe active nonatrophic gastritis due to helicobacter pylori, nonspecific chronic inflammation of duodenum and terminal ileum, and normal colonic mucosa. To rule out intestinal pathology, capsule endoscopy was performed which showed that diffuse polypoid lesions sized 2–5 mm throughout the small intestine was noted (Figure 3). Core biopsy of the liver showed peliotic changes, that is, blood filled cavities in the parenchyma not lined by endothelial cells (Figure 2(c)). Laparoscopic biopsies were taken from the omentum and parietal peritoneum. Omental biopsy analysis showed nonspesific mild chronic inflammatory infiltration, proliferation of mesothelial cells while peritoneal biopsy analysis revealed diffuse lymphangiectasia and papillary proliferation of mesothelial cells with vascular congestion (Figure 2(d)). Genetics consultation and karyotype analysis did not detect any pathology. The diagnosis was established as noncirrhotic portal hypertension, peliosis hepatis, portal vein aneurysm, and intestinal lymphangiectasia according to clinical, laboratory, radiological, and pathological findings. For assesment of lymph drainage, magnetic resonance (MR) lymphangiography was performed which showed significant dilatation of cisterna chili and saccular changings of lymphatic channels (Figures 1(b)-1(c)). 

Peritoneovenous shunt operation was performed due to diuretic resistant ascites and intolerance of frequent paracentesis. The patient was readmitted one week after surgery for fever and hyperemia at the subcostal and cervical incision sites. The condition was evaluted as graft infection. Chest and abdominal CT revealed total occlusion of 3 cm segment of internal jugular vein and a partial thrombus occluding 50% of the lumen through thoracic outlet at the level of the graft. Under fluoroscopy contrast media was injected through the shunt reservoir to show severe reduction of drainage from the graft to jugular vein. Diagnosis of shunt dysfunction was made and a catheter was placed to subclavian vein for bolus infusion of thrombolytic agent (tissue plasminogen activator, tPA). 24 hours after the procedure, tPA and low dose unfractionated heparin infusions were initiated. The infusion therapy had to be stopped at the 12th hour due to hematemesis and melena. Hemodynamic status of patient was stable after blood transfusions and fluid resuscitation. One week after the thrombolytic therapy, the thrombus was recanalized and a peritoneovenous catheter was reimplanted into subclavian vein. The patient was discharged 10 days after the application of the thrombolytic treatment. In the outpatient follow-up, there was no reduction in ascites and severe malnutrition due to malabsorption developed despite adequate enteral and parenteral nutrition therapy. The patient died of sepsis (nasocomial pneumonia—hemoculture positive for *Klepsiella pneumoniae*) 3 months after the surgery.

## 3. Discussion

Portal vein aneurysm (PVA) is a very rare vascular abnormality and up to date, there has been limited number of reported cases. However, it has been increasingly described recently probably because new imaging techniques are more available in clinical practice [1]. Ultrasonographic studies showed that the maximum anteroposterior diameter of the portal vein did not exceed 15 mm in normal subjects and 19 mm in those with cirrhosis. Aneurysm is diagnosed when anteroposterior diameter of the portal vein exceeds 20 mm [2]. Two forms, congenital and acquired, have been described. Congenital PVA is suspected in patients with no history of trauma, pancreatitis, liver biopsy, hepatic tumor, arterioportal fistula, or cirrhosis [3]. Congenital PVAs are mostly extrahepatic. In our case, the aneurysm was extrahepatic and ureteropelvic junction narrowing, well-known congenital abnormality, coexisted. Another interesting finding of our case is the association of the left gastric vein (coronary vein) aneurysm which was not reported previously with PVA. Taking together, we believe that PVA of our patient is congenital. 

Peliosis hepatis is a rare disorder characterized by the presence of cystic, blood-filled spaces of variable size in the liver [4]. Two forms—microscopic and macroscopic—have been described. In our patient, the liver was normal under US and CT imaging; the diagnosis of peliosis was made by liver biopsy. Regarding the etiology of peliosis hepatis, many hypotheses such as congenital malformation [5], androgenic anabolic steroid or oral contraceptive administration, and acquired immunodeficiency syndrome have been proposed [6, 7]. In our case, we could not find any etiologic factor to cause peliosis hepatis. Therefore we believe that peliosis of the case is idiopathic. The morphogenesis of peliosis is controversial. It has been attributed to increased sinusoidal pressure because of obstacles to blood outflow from the liver, the disappearance of normal parenchyma by necrosis of liver cells, and sinusoid wall weakness [7]. In our case, there was neither any difficulty in blood outflow from the liver and nor parenchymal necrosis. Therefore, it is likely that congenital sinusoid wall weakness by itself is the cause of peliosis in our case. 

Another finding of our patient was lymphangiectasia which was seen in peritoneum and small intestine. Lymphangiectasia is characterized by the presence of dilated lacteals resulting from an obstructive process of lymph drainage. Two forms of intestinal lymphangiectasia have been described—primary and secondary. The primary form results from the malformation of the lymphatic drainage system of gastrointestinal tract. Secondary lymphangiectasias are due to diseases that block the intestinal lymph drainage. These include retroperitoneal fibrosis, extensive retroperitoneal or abdominal tumors, Crohn's disease, Whipple's disease, celiac disease, constructive pericarditis, SLE, mesenteric tuberculosis or sarcoidosis, and chronic heart failure [8]. In our case obstruction of lymph drainage was demonstrated clearly by magnetic resorance lymphangiography (MRL). MRL showed saccular dilatations and narrowings of abdominal lymphatic vessels. We could not find any disorder to cause this pathology, therefore we belive that lymphangiectasia in our case is primary.

Analogous to portal vein aneurysms, sinusoidal and lymphatic dilatations may also be regarded as vascular malformations. Although there is no previous report in the literature, the associations of PVA have been reported to coexist with hereditary hemorrhagic telangiectasia [9], liver hemangiomas, intracranial arteriovenous malformation [10], and bowel venous malformation [11]. The histological examination of venous aneurysms demonstrates wall weakness with a decrease in the number and size of the elastic and muscle fibers of the vein's wall and fragmentation of the internal elastic layer, with replacement by fibrous connective tissue [12]. If we assume that wall weakness exists in all malformations present in our case, it can be speculated that an undefined genetic mutation related to the development of vascular wall is responsible for presence of PVA, PH, and IL. Unfortunately, we could not prove this hypothesis.

## 4. Conclusion

Portal vein aneurisym (PVA), peliosis hepatis (PH), and intestinal lymphangiectasia (IL) all are very uncommon entities. Herein, we presented a unique patient with these three rare entities who was admitted to our hospital because of portal hypertensive ascites rich in protein and lymphocyte. PVA was extrahepatic and associated with coronary vein aneurysm. Peliosis hepatis was of microscopic form. Lymphangiectasia was present in peritoneum and small intestine. Diagnoses of these rare entities were made by imaging techniques and histopathological findings. Patient also had hydronephrosis caused by ureteropelvic junction narrowing. As far as our knowledge from the literature, there is no such a case reported previously with the association of PVA, PH, and IL. Therefore, we propose PVAPHIL syndrome to define this novel association.
